# Successful and unsuccessful weight-loss maintainers: strategies to counteract metabolic compensation following weight loss

**DOI:** 10.1017/jns.2018.11

**Published:** 2018-06-28

**Authors:** Louise D. Clamp, David J. Hume, Estelle V. Lambert, Jacolene Kroff

**Affiliations:** Division of Exercise Science and Sports Medicine, Department of Human Biology, Faculty of Health Sciences, University of Cape Town, South Africa

**Keywords:** Energy expenditure, Substrate utilisation, Weight-loss maintenance, Weight-loss relapse, FFM, fat-free mass, FM, fat mass, LSW, low-weight stable weight, NREE, non-resting energy expenditure, OSW, overweight/obese stable weight, RED, reduced-overweight/obese, REL, relapsed-overweight/obese, TDEE, total daily energy expenditure, TDEI, total daily energy intake, TEF, thermic effect of feeding

## Abstract

Adaptive thermogenesis and reduced fat oxidative capacity may accompany weight loss, continuing in weight maintenance. The present study aimed (1) to determine whether weight-reduced and weight-loss relapsed women are at greater metabolic risk for weight gain compared with BMI-matched controls with no weight-loss history, and (2) to identify protective strategies that might attenuate weight loss-associated adaptive thermogenesis and support successful weight-loss maintenance. Four groups of women were recruited: reduced-overweight/obese (RED, *n* 15), controls (low-weight stable weight; LSW, *n* 19) BMI <27 kg/m^2^; relapsed-overweight/obese (REL, *n* 11), controls (overweight/obese stable weight; OSW, *n* 11) BMI >27 kg/m^2^. Body composition (bioelectrical impedance), 75 g oral glucose tolerance test, fasting and postprandial metabolic rate (MR) and substrate utilisation (RER) and physical activity (accelerometer (7 d)) were measured. Sociobehavioural questionnaires and 3 × 24 h diet recalls were completed. Fasting and postprandial MR, RER and total daily energy intake (TDEI) were not different between RED and REL *v.* controls (*P* > 0·05). RED consumed less carbohydrate (44·8 (sd 10·3) *v.* 53·4 (sd 10·0) % TDEI, *P* = 0·020), more protein (19·2 (sd 6·0) *v.* 15·6 (sd 4·2) % TDEI, *P* = 0·049) and increased physical activity, but behaviourally reported greater dietary restraint (*P* = 0·002) compared with controls. TDEI, macronutrient intake and physical activity were similar between OSW and REL. REL reported higher subjective fasting and lower postprandial ratings of prospective food consumption compared with OSW. Weight-reduced women had similar RMR (adjusted for fat-free mass) compared with controls with no weight-loss history. Increased physical activity, higher protein intake and greater lean muscle mass may have counteracted weight loss-associated metabolic compensation and highlights their importance in weight-maintenance programmes.

Global obesity rates are estimated to reach 10·8 % in men and 14·9 % in women, carrying increased risk for health outcomes^(^^1^^–^^3^^)^. Small degrees of weight loss reduce obesity-associated chronic disease risk^(^^4^^)^; however, weight loss is difficult to achieve and maintain^(^^5^^–^^7^^)^. None-the-less, successful weight-loss maintenance is possible^(^^8^^,^^9^^)^ and evidence suggests that if weight loss can be maintained for 2–5 years, the chances of relapse are greatly reduced^(^^10^^–^^12^^)^.

Adaptive thermogenesis describes the reduction in total daily energy expenditure (TDEE) (including RMR and non-resting energy expenditure (NREE)) in response to energy deficit, beyond that predicted by changes in body composition, and is implicated in a reduced capacity for ongoing weight loss as well as weight-loss relapse^(^^13^^–^^16^^)^. While slower rates of weight loss allow for greater loss of fat mass (FM) and preservation of fat-free mass (FFM), this does not reduce the adaptive decline in RMR following 10 % weight loss^(^^17^^)^. With an initial 10 % weight loss, RMR and NREE show similar declines, while with a further 10 % subsequent weight loss the adaptive response is lower, predominantly affecting NREE^(^^18^^)^. Lower RMR predicts future weight gain independent of BMI or baseline body composition^(^^15^^,^^17^^,^^19^^,^^20^^)^. Metabolically active FFM (organs and skeletal muscle) does contribute significantly to RMR^(^^21^^)^. Greater body fat percentage loss, and sparing of FFM during weight loss lower body-weight regain over the following 2 years^(^^22^^)^. However, in weight-loss maintenance at 1-year follow-up, decreases in TDEE, NREE and to a lesser extent RMR are shown and correlate with weight loss^(^^23^^,^^24^^)^. Given that decreases in all components of energy expenditure can be expected in individuals with a history of weight loss, it would be of importance to establish whether this would distinguish them from phenotypically similar individuals with no weight-loss history, increasing their risk for future weight gain.

Metabolic flexibility is the ability to transition between fat and carbohydrate oxidation depending on availability^(^^25^^)^. Metabolic inflexibility increases with obesity and insulin resistance, reducing both fasting fat oxidation and carbohydrate oxidation under insulin-stimulated conditions^(^^25^^)^. Formerly obese individuals have shown lower fasting and postprandial fat oxidation responses compared with matched controls^(^^26^^,^^27^^)^. Low fasting fat oxidation increases relative carbohydrate utilisation, thus favouring fat storage over oxidation and increasing the risk for greater fat deposition, particularly at times of positive energy balance on dietary relapse^(^^6^^,^^28^^–^^34^^)^. Suppressed fat oxidation accompanying prior weight loss would therefore be likely to increase the risk for future weight gain and fat deposition relative to individuals who had never engaged in periods of weight loss.

Weight loss-associated declines in components of metabolism combined with suppressed fat oxidation, at rest and postprandially, predispose weight-reduced individuals to increased fat storage and subsequent weight regain. We hypothesised that, in light of metabolic changes accompanying weight loss, weight-reduced and weight-loss relapsed individuals would exhibit reduced energy expenditure and fat oxidative capacity compared with phenotypic controls with no weight-loss history, potentially predisposing them to subsequent weight gain. The primary aim of the present study was to compare fasting and postprandial metabolic rate and substrate utilisation, both absolute and per kg FFM, along with subjective appetite ratings between (1) successfully maintained, weight-reduced individuals; (2) weight-loss relapsed individuals; and their respective BMI-matched controls with no weight-loss history, to establish whether weight-loss history increases their risk for future weight gain. The secondary aim was to compare behavioural factors (dietary intake, eating behaviour and physical activity) and body composition (secondary outcomes) between these groups and to explore their association with RMR, to assess whether these contribute to, or counteract metabolic adaptions to weight loss.

## Methods

### Subject selection and screening

Advertisements were placed at local institutions and on the Sport Science Institute of South Africa (SSISA) website, stipulating that previous weight loss had to be intentional/deliberate, without the use of unregulated products, through lifestyle-related approaches (diet and/or exercise), unrelated to stress and/or anxiety and free of eating pathology. Participants were screened and placed into four groups. Successful weight reduction is defined as weight loss of ≥10 %, maintained for over 12 months with weight fluctuations of 3 % considered acceptable^(^^35^^,^^36^^)^. Successfully reduced (RED) individuals recruited had lost ≥15 % of body weight from a BMI ≥27 kg/m^2^, maintained weight for over 12 months with 12-month fluctuations of ≤5 %. Weight-relapsed (REL) individuals (BMI ≥27 kg/m^2^) had previously lost ≥15 % of body weight and regained most/all of this weight. Age- and BMI-matched, low-weight stable weight (LSW) controls (BMI ≤27 kg/m^2^) and overweight/obese stable weight controls (OSW) (BMI ≥27 kg/m^2^) had no prior weight-loss history. Exclusion criteria included being pregnant or lactating, irregular menstrual cycles (<7 cycles per year/cycle intervals >35 d), medical condition and/or condition requiring chronic medication that affected metabolic rate (B2-agonists, β-blockers, corticosteroids, etc.), weight-loss medication/supplements, diagnosis of thyroid dysfunction or an eating disorder.

The study protocol was approved by the University of Cape Town Faculty of Health Science and Human Research Ethics Committee (HREC 214/2012). Prior to testing, all participants were given full information of test procedures, signed informed consent forms and were at liberty to withdraw at any time.

### Experimental trial

**Fig. 1.**
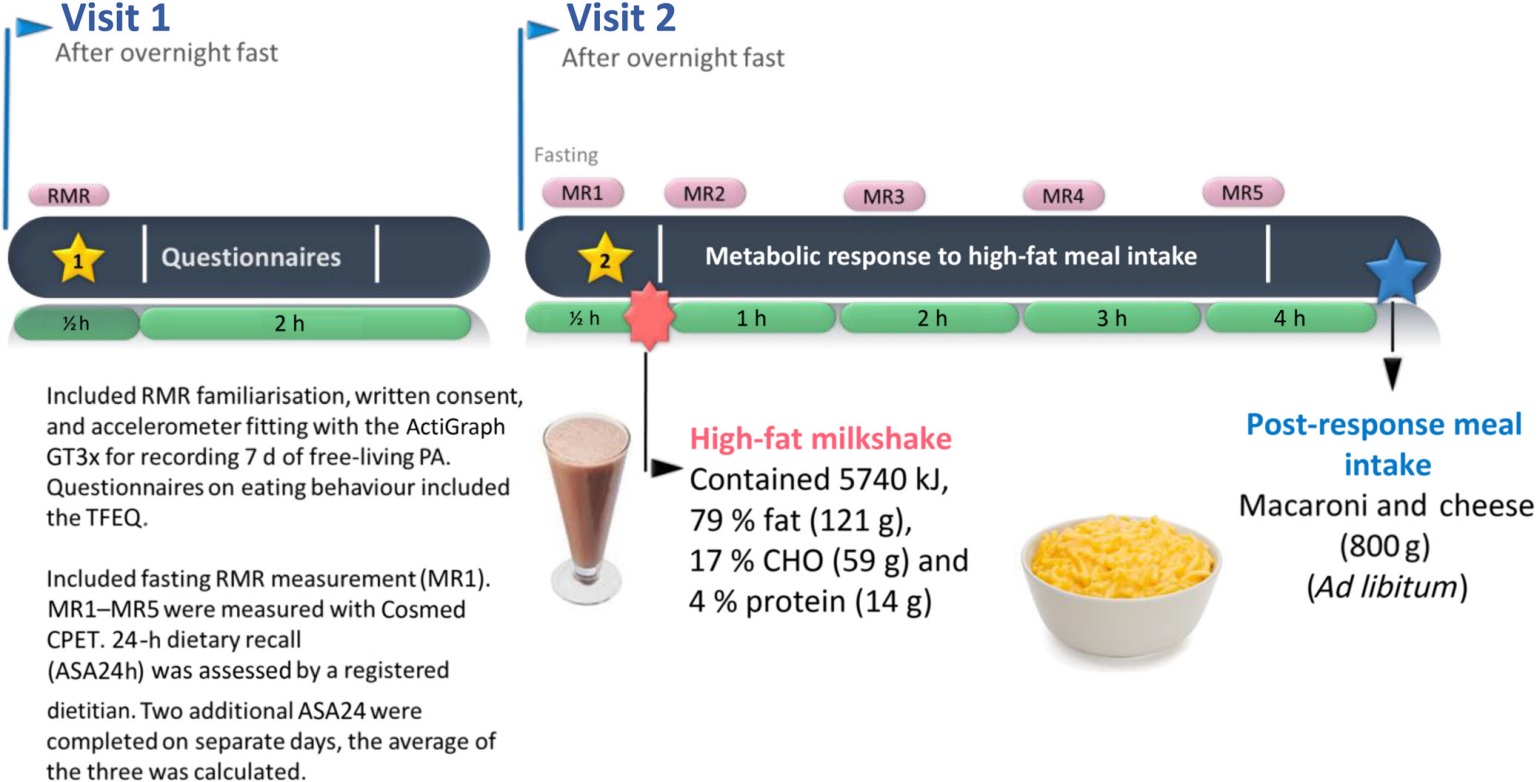
Experimental trial. PA, physical activity; TFEQ, Three Factor Eating Questionnaire; CHO, carbohydrate.

Participants attended two laboratory visits (see Fig. 1). During the first visit participants reported in the morning fasted, following an overnight fast (10–12 h). RMR and substrate utilisation, body composition, heart rate and blood pressure were measured. An oral glucose tolerance test was conducted during which time participants completed questionnaires covering socio-economic status, education, psychological status, stress and eating behaviour including the Three Factor Eating Questionnaire (factor I representing dietary restraint; factor II representing disinhibition of control; and factor III representing hunger)^(^^37^^)^. They were then fitted with accelerometers (ACTi Graph; Shalimar) to record physical activity over a 7 d period.

At the second visit, participants reported to the laboratory in the morning, fasted following an overnight fast (10–12 h). Fasting RMR (MR1), RER and subjective appetite ratings were measured. Participants then ingested a standardised high-fat test meal over 10 min. Immediately thereafter, RMR, RER and appetite ratings were measured (MR2) and repeated every 1 h for 3 h (MR3–5). After 4 h, appetite ratings were measured and participants consumed a self-selected portion of a standardised lunch and completed the final appetite rating. The amount consumed was covertly measured by weighing before and after portion selection. Participants remained resting, but awake for the duration of the trial, were permitted to watch videos and could drink 250 ml of water. With a registered dietitian, participants completed one automated, online, self-administered 24-h dietary recall (ASA24™, Applied Research Program, National Cancer Institute)^(^^38^^)^ and recorded food intake on two additional days.

### Anthropometry

Fasted and prior to testing, weight (BW-150; NAGATA), height (3PHTROD-WM; Detecto), and waist and hip circumferences were measured. Body composition was measured using bioelectrical impedance analysis (BIA) (Quantum II; RJL Systems). Participants refrained from drinking water 4 h prior to the BIA measurement and were requested to void their bladder.

### Metabolic rate measurements and calculations

For both laboratory visits fasting RMR and RER were measured in the morning following a 10–12 h overnight fast. Reported fasting measures used are those recorded on the second test visit. Participants rested in the supine position, in a temperature-controlled (21–24°C) room. RMR and RER were measured for 20 min, using the ventilated hood technique (Cosmed Quark CPET)^(^^39^^)^. During the second visit this was repeated immediately after consumption of the test meal and repeated every 1 h thereafter for 3 h (i.e. 20 min measurement followed by a 40 min rest). Prior to testing the metabolic cart was calibrated with a Hans Rudolph 3L syringe and analysers calibrated using normal room air (21 % O_2_, 4 % CO_2_, balance N_2_) and standard gas mixtures (5 % CO_2_, 16 % O_2_, balance N_2_) (BOC Special Gas). RMR, and total fat and carbohydrate oxidation were calculated using the equations of Weir^(^^40^^)^ and Frayn^(^^41^^)^, respectively. Thermic effect of feeding (TEF) was calculated as AUC for energy expenditure after the test meal, expressed as the percentage increase in RMR in response to the test meal (i.e. increase in RMR resulting from processes of digestion, absorption, metabolism, storage and/or elimination of ingested nutrients and/or substrates)^(^^42^^)^. This increase in RMR has also been expressed in absolute terms (postprandial energy expenditure) over the test period as well as relative to FFM. Postprandial energy balance was calculated as the difference between energy ingested in the test meal and postprandial energy expenditure. Postprandial fat balance was calculated as the difference between fat ingested in the test meal and fat oxidised in the postprandial period.

### 75 g Oral glucose tolerance test blood sampling and analysis

At visit 1 following fasting RMR measurements, a cannula attached to a three-way stopcock was inserted into the antecubital vein. Fasting blood samples (about 5 ml) were drawn to determine fasting plasma glucose and insulin. A 75 g glucose solution was consumed and blood samples collected at 2 h. Samples were kept on ice until centrifuged at 3000 rpm at 4°C for 10 min and subsequently stored at −80°C for later analysis. Plasma glucose concentrations were determined using the glucose oxidase method (YSI 2300 STAT PLUS; YSI Life Sciences) and serum insulin by direct chemiluminescence immunoassay using the Centaur CPImmunoassay System (Siemens Medical Solutions Diagnostics). Insulin sensitivity was estimated using the homeostasis model assessment for insulin resistance (HOMA-IR)^(^^43^^)^.

### Test meal

At visit 2 following fasting measurements, a liquid test meal was consumed (milkshake: 5743 kJ (1372 kcal), 200 ml cream, 200 g full-fat ice-cream, 100 ml full-cream milk; 79 % fat (120 g), 17 % carbohydrate (59 g), 4 % protein (14 g)), reflecting an energy-dense food available to free-living individuals. Liquid test meals have also been shown to have high reproducibility for postprandial energy expenditure^(^^44^^)^. The high-fat content of the test meal was chosen to optimise detection of metabolic and appetite differences between groups in response to dietary fat. Obese individuals have been shown to be prone to passive overconsumption following energy-dense, high-fat meals while lean individuals are resistant to this^(^^45^^)^. All participants consumed a fixed volume of the standardised test meal, replicating the approach of earlier studies^(^^46^^,^^47^^)^ and to examine differences in physiological response to the same energy-dense meal.

### Post-test meal

At 4 h after the test meal, participants consumed a self-selected quantity of a standardised lunch (ready-made macaroni cheese: 577 kJ (138 kcal) per 100 g, 35 % fat, 48 % carbohydrate and 17 % protein; Woolworth's, South Africa). The container was weighed before and after portion selection and plate wastage measured to determine amount consumed. Time taken to complete the post-test meal was recorded.

### Subjective appetite measurement

Subjective ratings of appetite (hunger, fullness, desire to eat, prospective food consumption (PFC) and satiety) were recorded before, immediately after the test meal, every 1 h thereafter for 4 h and again following the post-test meal. A validated visual analogue scale was used^(^^48^^)^. Participants placed a mark along a 100 mm line representing their subjective rating for each appetite variable, anchored with the opposing extremes of the sensation (e.g. ‘not at all full’ (0 mm) to ‘extremely full’ (100 mm)).

### Dietary intake – automated self-administered 24 h recall (ASA24)

Dietary intake data were recorded and analysed using the validated, automated online self-administered 24-h dietary recall (ASA24™; Applied Research Program, National Cancer Institute) based on the automated multiple-pass method^(^^38^^)^. A registered dietitian guided participants through the first 24 h recall and completed 2 further days. Two weekdays and one weekend day were recorded to control for variation in intake. The online ASA24 software performs well against interviewer-administrated 24-h recall and in comparison with actual energy and macronutrient intake^(^^49^^)^.

### Objectively measured physical activity

ACTi Graph GT3X (ACTi Graph) tri-axial accelerometers, fitted at the first laboratory visit, recorded habitual physical activity. The device was worn on the right hip for seven consecutive days and removed only during night-time sleep, bathing, showering and swimming. A minimum of 4 d and 600 min per d was required for data analysis as provides 80 % reliability^(^^50^^)^. Accelerometer data were downloaded and exported to Excel data tables using ACTi Life Software Version 5 and analysed for light, moderate and vigorous activity occurring in 1 min count intervals^(^^51^^)^. Light, moderate and vigorous intensity physical activity levels were estimated based on accelerometer counts using cut-points according to Matthews^(^^52^^)^. The Matthews equation stipulates counts between 101 and 759 (equivalent to 2–2·9 metabolic equivalents of task (MET)) represent light-intensity physical activity, counts between 760 and 5998 (equivalent to 3–6 MET) represent moderate-intensity physical activity, and counts above 5999 indicate vigorous activity^(^^52^^)^. Energy expenditure was calculated using the physical activity level method^(^^53^^)^.

### Statistical analysis

Sample size determination was based on previous studies in which mean differences between lean controls and weight-reduced individuals in RER of 0·05 (sd 0·04) and RMR 891 (sd 824) kJ (213 (sd 197) kcal) were observed^(^^27^^,^^54^^)^. Using a power of 80 % and an α level of 0·05 an estimated sample size was 8–18 women per group. Data were assessed for normality using histogram plots and the Shapiro–Wilk's test, where *P* < 0·05 indicated that data were not normally distributed. Means and standard deviations were reported for normally distributed data and medians and interquartile ranges for non-parametric data. This analysis compares RED and REL against their respective phenotypic controls to identify whether, following either weight loss or subsequent weight regain, these individuals return to being indistinguishable from phenotypically similar counterparts with no weight-loss history. Therefore, two group comparisons of normally distributed data were carried out, using two-sample *t* tests for independent groups with equal variance or a Satterthwaite's independent-samples *t* tests for unequal variance. Non-parametric data used Wilcoxon rank-sum tests. Mixed models with repeated measures were used to compare differences in the changes in subjective ratings of appetite between groups. In line with the primary aim of the present study (i.e. to compare BMI-matched individuals), differences between RED *v.* LSW controls, and REL *v.* OSW control participants were explored. Pearson's correlation coefficient was used to test for associations between RMR and FFM, both being normally distributed. For all tests significance was accepted at *P* < 0·05.

## Results

### Participant characteristics, body composition and weight history

There were no differences in body fat percentage, FM and waist circumference; however, RED had greater FFM (*P* = 0·018) and lower waist:hip ratio (*P* = 0·052) compared with LSW (Table 1). RED had lost 16·1 % (14·4–22·0 %) of body weight (BW), maintained for 30 months (12–60 months). REL had lost 19·1 % (17·3–29·5 %) of BW and subsequently regained 21·0 % (15·4–26·7 %) of BW.

**Table 1.** Participant characteristics and weight history (Median values and interquartile ranges (IQR); mean values and standard deviations)

	LSW (*n* 19)		RED (*n* 15)		OSW (*n* 11)		REL (*n* 11)	
	Mean	sd	Mean	sd	Mean	sd	Mean	sd
Age (years)								
Median	28		32		32		34	
IQR	25–37		26–40		29–40		22–41	
BW (kg)								
Median	59·9		67·1		87·6		92·5	
IQR	56·8–68·3		61·5–74·0		83·4–96·0		79·1–103·3	
BMI (kg/m^2^)	22·7	2·3	24·1	2·3	35·0	4·1	34·1	6·2
Body fat (%)	29·1	5·2	29·6	4·3	45·4	4·1	43·4	6·6
FM (kg)	18·4	5·2	20·4	5·3	41·6	10·0	42·0	13·5
FFM (kg)	44·0	3·8	47·6*	4·8	49·1	5·3	52·8	5·7
Waist (cm)	68·4	4·8	69·4	5·4	91·8	9·0	90·7	9·6
Waist:hip ratio								
Median	0·69		0·66		0·78		0·75	
IQR	0·68–0·71		0·64–0·69		0·74–0·83		0·73–0·81	
Weight history								
Weight loss (% BW lost)†								
Median	−2·8		−16·1		−1·6		−19·1	
IQR	−4·5 to 0·0		−22·0 to −14·4		−3·4 to 0·0		−29·5 to -17·3	
Weight regain (% BW regain)‡								
Median	0·0		0·0		0·0		21·0	
IQR	0·0–1·5		0·0–2·8		1·1–2·7		15·4–26·7	
Time at current weight (months)								
Median	24		30		9·0		6·0	
IQR	12–60		12–60		6·0–12		3–30	

LSW, low-weight stable weight; RED, reduced-overweight/obese; OSW, overweight/obese stable weight; REL, relapsed-overweight/obese; BW, body weight; FM, fat mass; FFM, fat-free mass.

* *P* < 0·05 for two group comparisons of RED or REL with respective BMI-matched controls.

† Weight loss (% BW lost) = (heaviest adult weight – lightest subsequent weight)/(heaviest adult weight) × 100 %. In the case of RED and REL this would be heaviest weight prior to intended weight loss.

‡ Weight regain (% BW regain) = (lightest subsequent weight – current weight)/(lightest subsequent weight) × 100.

### Metabolic measurements: fasting and postprandial response

Although fasting and 2 h plasma glucose levels were similar, the RED had lower fasting insulin concentrations and were more insulin sensitive than LSW (Table 2). LSW had lower fasting RMR compared with RED, but adjusted for FFM there were no differences between low-weight and overweight/obese groups. The standard test meal represented a higher energy (131·4 (sd 10·9) *v.* 121·8 (sd 12·6) kJ/kg FFM, *P* = 0·021) and fat (2·8 (sd 0·2) *v.* 2·6 (sd 0·3) g/kg FFM, *P* = 0·021) intake per kg FFM and energy intake as a percentage of measured TDEE (94·8 % (94·4–95·1 %) *v.* 94·4 % (93·9–94·7 %), *P* = 0·015) in LSW compared with the RED, but was not different in comparisons of OSW *v.* REL (*P* = 0·137) for both energy and fat intake per kg FFM and for energy intake % TDEE (*P* = 0·248). However, TEF, postprandial energy expenditure (absolute and per kg FFM), postprandial energy balance, RER, fat oxidation rate and postprandial fat balance were similar between low-weight groups and between overweight/obese groups (*P* > 0·05).

**Table 2.** Metabolic measurements (Median values and interquartile ranges (IQR); mean values and standard deviations)

	LSW (*n* 19)		RED (*n* 15)		OSW (*n* 11)		REL (*n* 11)	
	Mean	sd	Mean	sd	Mean	sd	Mean	sd
Insulin sensitivity								
Fasting plasma insulin (mIU/l)								
Median	7·6		4·5*		12·4		10·8	
IQR	4·8–10·5		2·9–5·2		9·1–16·2		5·6–14·9	
Fasting plasma glucose (mmol/l)†								
Median	4·8		4·9		5·4		5·2	
IQR	4·6–5·1		4·5–5·2		4·8–6·2		4·7–7·5	
2 h plasma glucose (mmol/l)†								
Median	6·1		6·2		7·9		7·7	
IQR	5·3–7·3		5·3–6·7		6·7–8·9		6·1–9·8	
HOMA-IR								
Median	1·86		0·85*		3·10		2·36	
IQR	1·01–2·43		0·64–1·25		2·34–4·45		1·91–3·73	
Fasting energy and substrate metabolism								
RMR (kJ/d)	5954	619	6427*	732	6352	1071	6619	1289
RMR/kg FFM (kJ/kg FFM per d)	135·6	13·0	135·1	10·0	134·3	19·2	130·5	10·9
RER	0·76	0·76	0·78	0·04	0·78	0·03	0·79	0·04
Postprandial energy and substrate metabolism								
TEF (%)	16·4	6·3	13·4	6·9	13·7	9·6	13·4	8·9
Postprandial EE (kJ)‡	1008	100	1063	146	1054	197	1121	176
Postprandial EE (kJ/kg FFM)	23·0	2·1	22·2	2·5	21·8	4·2	21·3	2·5
Postprandial energy balance (kJ)‡	4732	100	4678	146	4686	197	4619	176
Postprandial RER‡	0·74	0·05	0·75	0·04	0·75	0·04	0·76	0·04
Fat oxidation (mg/min)								
Median	112·1		108·6		96·4		103	
IQR	78·0–129·0		84·8–124·5		78·0–154·2		86·6–126·5	
Postprandial fat balance (g)								
Median	97·0		98·4		100·3		98·9	
IQR	93·4–104·1		94·3–102·7		88·1–104·1		93·9–103·6	
Post-test meal								
Amount eaten (kJ)								
Median	1481		1435		1925		1460	
IQR	1038–2159		992–1887		1293–2222		1042–2397	
Amount eaten (% total daily EE)								
Median	24·6		24·1		32·7		26·3	
IQR	17·2–35·5		16·1–31·2		24·2–36·7		18·9–36·9	
Time taken (min)	10·9	4·8	10·4	3·4	11·4	4·4	7·06*	2·2

LSW, low-weight stable weight; RED, reduced-overweight/obese; OSW, overweight/obese stable weight; REL, relapsed-overweight/obese; HOMA-IR, homeostatic model assessment for insulin resistance; FFM, fat-free mass; TEF, thermic effect of feeding; EE, energy expenditure.

* *P* < 0·05 for two group comparisons of RED or REL to respective BMI-matched controls.

† Numbers with impaired fasting glucose (fasting glucose ≥5·6 mmol/l): LSW 2/19, RED 3/15, OSW 5/11 and REL 5/11. Numbers with impaired glucose tolerance (2 h glucose ≥7·8 mmol/l): RED 1/15, LSW 4/19, OSW 5/11 and REL 6/11.

‡ Postprandial EE and RER were calculated as the AUC of hourly metabolic measurements following ingestion of the test meal. Postprandial energy balance was calculated as the energy intake at the test meal minus postprandial EE; fat balance was calculated as the fat intake at the test meal minus postprandial fat oxidation.

### Subjective appetite response

**Fig. 2.**
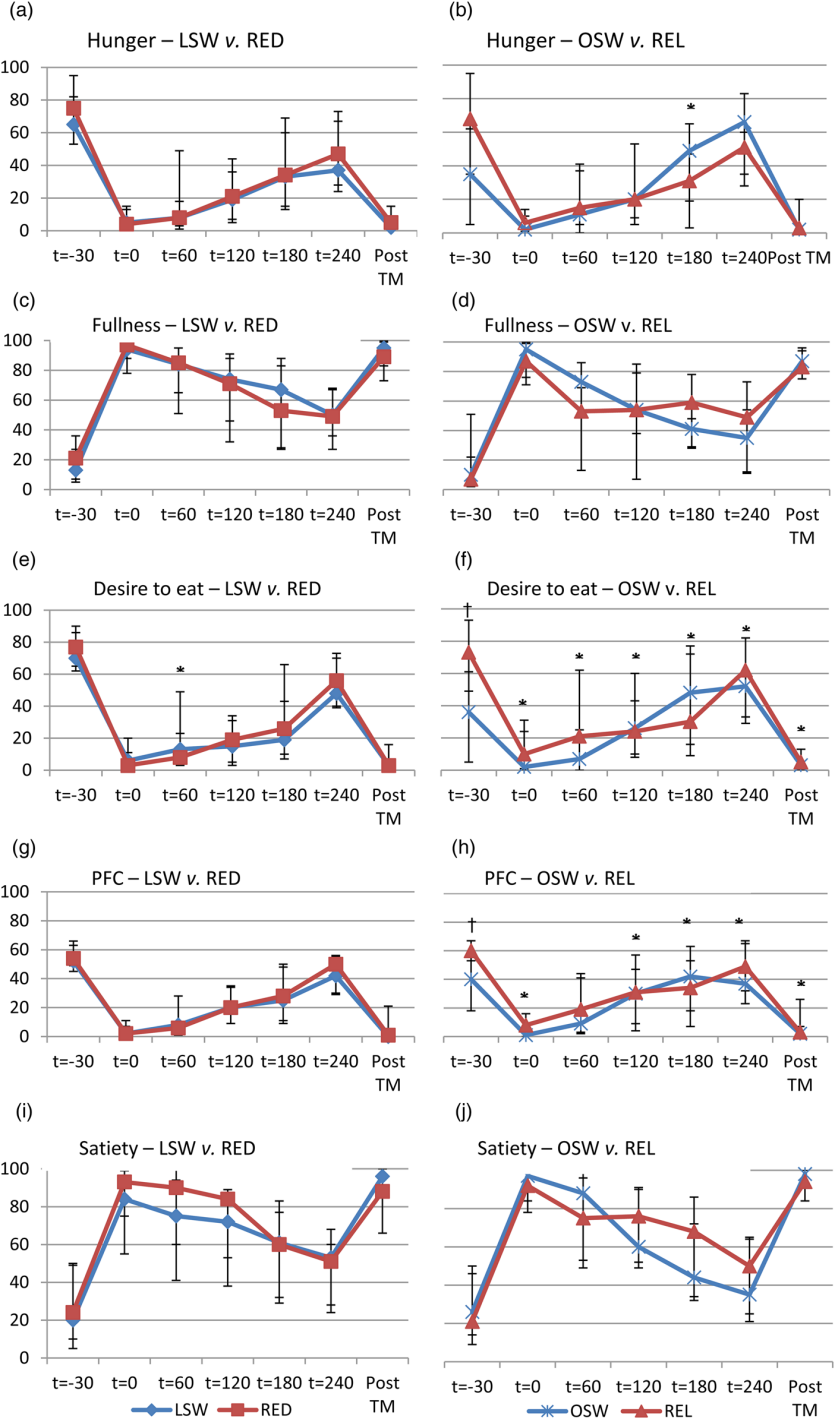
Subjective appetite measurements (mm) for low-weight stable weight *v.* reduced-overweight/obese (LSW *v.* RED) groups (left column) and overweight/obese stable weight *v.* relapsed-overweight/obese (OSW *v.* REL) groups (right column). The time points are as follows: *t* = −30 min represented the fasting measurement; *t* = 0 was the measurement immediately after consumption of the test meal; *t* = 60, *t* = 120, *t* = 180 and *t* = 240 were the measures recorded every 1 h after consumption of the test meal; post-TM was the subjective ratings recorded after *ad libitum* consumption of the post-test meal. PFC, prospective food consumption. Values are medians, with interquartile ranges represented by vertical bars. * Group × time effect (*P* < 0·05). † Group difference (*P* < 0·05).

For subjective appetite measures there were no group differences between LSW and RED (Fig. 2, left column); however, the change in ‘desire to eat’ from the fasted state to 60 min post-ingestion showed a greater decline in RED (group × time; *P* = 0·033). For the overweight/obese groups, ‘desire to eat’ (Fig. 2(f)) and ‘PFC’ (Fig. 2(h)), REL show higher subjective ratings in the fasted state (group differences *P* = 0·003 and *P* = 0·023, respectively), which subsequently decline and remain below fasted levels for the remainder of the trial. OSW, however, show lower ratings in the fasted state, but the suppression of these ratings, post-ingestion of the test meal, is less pronounced than for REL (group × time; *P* < 0·05) and at 180 min these ratings return to baseline or above fasting levels.

### Post-test meal

LSW and RED consumed similar amounts of the post-test meal, taking comparable times to complete the meal. While REL and OSW groups consumed similar quantities of food, REL consumed the post-test meal faster than OSW (*P* = 0·015).

### Dietary intake and physical activity

Total daily energy intake (TDEI) for RED and REL did not differ from respective controls (Table 3). REL tended to report lower TDEI compared with controls (*P* = 0·076) and this became significant when adjusted for FFM (*P* = 0·017). RED consumed less carbohydrate and more protein compared with controls (*P* < 0·05), and had a tendency for higher fat intake (*P* = 0·053). They also reported higher scores for dietary restraint (*P* = 0·002) with a tendency for periods of disinhibited eating (*P* = 0·076). REL and OSW had similar macronutrient composition and eating behaviour scores. Although statistically significant, TDEE was only marginally higher than LSW; however, they were less sedentary, did more light (*P* = 0·024) and moderate activity (*P* = 0·032) and a tendency for greater vigorous activity (*P* = 0·061). REL and OSW had similar levels of TDEE. None of the groups was in energy deficit (TDEI:TDEE ratios), although this ratio was higher in OSW compared with REL (*P* = 0·005).

**Table 3.** Energy intake (EI) and energy expenditure (EE) (Median values and interquartile ranges (IQR); mean values and standard deviations)

	LSW (*n* 19)		RED (*n* 15)		OSW (*n* 11)		REL (*n* 11)	
	Mean	sd	Mean	sd	Mean	sd	Mean	sd
Dietary intake and eating habits								
Total daily EI (kJ/d)								
Median	6945		6473		9104		6577	
IQR	5427–7912		5791–8619		8180– 11 673		6284–9615	
Total daily EI per kg FFM (kJ/kg FFM)								
Median	149·4		149·0		188·7		136·0*	
IQR	125·5–170·3		121·8–179·1		167·8–236·4		114·6–174·9	
Fat (% total daily EI)	32·1	6·9	36·9	6·8	40·3	7·0	35·4	8·8
Protein (% total daily EI)	15·6	4·2	19·2*	6·0	17·4	4·0	19·7	4·2
Protein (g/kg)	0·96	0·26	1·15*	0·23	1·11	0·33	1·01	0·34
Carbohydrate (% total daily EI)	53·4	10·0	44·8*	10·3	42·8	8·4	44·8	9·4
TFEQ-factor I (restraint)	7·7	4·1	12·1*	3·6	7·0	3·8	9·6	4·8
TFEQ-factor II (disinhibition)	5·8	3·9	8·3	3·9	9·5	3·7	11·1	4·2
TFEQ-factor III (hunger)	4·8	3·2	5·7	3·7	8·5	3·5	7·4	3·6
Physical activity								
Sedentary/light (kJ/d)								
Median	5874		5786*		5895		5812	
IQR	5791–5975		5586–5866		5849–5920		5619–5933	
Moderate (kJ/d)								
Median	142		251*		159		255	
IQR	59–251		146–402		126–205		109–402	
Vigorous (kJ/d)								
Median	0		42		0		0	
IQR	0–21		0–105		0–4		0–0	
Total daily EE (kJ/d)†								
Median	6054		6079*		6050		6067	
IQR	6033–6084		6058–6113		6046–6058		6046–6088	
Energy balance								
Total daily EI:EE†	1·08	0·23	1·18		1·53		1·10*	0·10

LSW, low-weight stable weight; RED, reduced-overweight/obese; OSW, overweight/obese stable weight; REL, relapsed-overweight/obese; FFM, fat-free mass; TFEQ, Three Factor Eating Questionnaire.

* *P* < 0·05 for two group comparisons of RED or REL to respective BMI-matched controls.

† Determined from ActiGraph measurements.

### Associations with RMR

FFM was positively correlated with RMR for the overall group (*r*^2^ 0·572; *P* < 0·001) as well as for RED (*r*^2^ 0·765; *P* < 0·001) and REL (*r*^2^ 0·634; *P* = 0·036) and approached significance for LSW (*r*^2^ 0·425; *P* = 0·070), but showed no association in OSW. Both TDEE and TDEI were positively associated with RMR (*r*^2^ 0·304, *P* = 0·030 and *r*^2^ 0·289, *P* = 0·031, respectively). None of the macronutrient intakes (% TDEI) was associated with RMR; however, the association between RMR and protein intake in g/d in RED approached significance (*r*^2^ 0·498; *P* = 0·058).

## Discussion

The main findings of the present study were that weight-reduced women had similar RMR (adjusted for FFM) and substrate utilisation compared with controls with no weight-loss history. Nevertheless, successful weight-loss maintainers showed behavioural differences that, together, might have counteracted metabolic adaptations to weight loss. These included subjectively indicating greater discipline around dietary intake, reporting increased protein intake and reduced carbohydrate intake, being more physically active and having greater FFM. However, maintaining reduced weight remains challenging over the longer term. Disciplined behaviour, as well as the employment of strategies that bolster both resting energy expenditure and NREE, are undoubtedly important in reducing metabolic risk for weight regain and supporting successful weight-loss maintenance. Weight-loss relapsed individuals showed no differences in metabolic measurements, but subjectively reported increased appetite in the fasted state. However, following ingestion of a high-fat test meal, they reported greater reductions in these measures compared with obese/overweight individuals who had never attempted weight loss, suggesting greater drive to consume food in the fasted state but also increased sensitivity to moderating this drive postprandially.

Previously studies have examined energy balance components in order to identify the energy gap that might both explain causes of obesity and help to identify potential remedial action^(^^55^^–^^57^^)^. TDEE is comprised of RMR (about 50–70 %), TEF (about 5–15 %) and NREE (planned and spontaneous physical activity)^(^^58^^)^. Moderate energy restriction, exercise and maintenance of FFM helps to attenuate the decline in RMR accompanying weight loss; however, extreme energy restriction, even when combined with exercise and maintenance of FFM, results in significant declines in RMR that persist for prolonged periods^(^^24^^,^^58^^–^^60^^)^. Other studies have found that this adaptive response associated with energy restriction reflects a ‘transient hypothyroid hypo-metabolic state’ that normalises once energy balance is re-established^(^^61^^)^. In this study, weight-reduced individuals successfully maintained goal weight for a median of 30 months. Furthermore, none of the groups studied was in energy deficit and were weight stable at the time of testing. Weight-reduced individuals in this study reported consuming more dietary protein compared with controls and this was found to be positively associated with both FFM and RMR, specifically in RED, confirming findings of previous studies^(^^62^^,^^63^^)^. RED also reported consuming less carbohydrate compared with LSW, and similar higher-fat, lower-glycaemic load weight-maintenance diets have been shown to result in smaller declines in energy expenditure^(^^64^^)^. Taken together with the fact that RED had greater FFM and were generally more physically active, these factors may explain the lack of relative metabolic disadvantage as a result of prior weight loss. This highlights the importance of incorporating similar strategies within weight-maintenance programmes to reduce the impact of adaptive thermogenesis and assist in successfully maintaining reduced weight.

TEF, measured as the percentage increase in RMR following ingestion of a meal, was similar between RED and REL compared with their respective controls. While some studies have reported reductions in TEF following weight loss and stabilisation over shorter time periods of 10 d^(^^28^^)^, these results show no indication of this in RED or REL groups. It has also been suggested that the TEF is associated with appetite or satiety, although a recent meta-analysis found no evidence to support this^(^^42^^)^. Appetite ratings, both fasted and postprandial, are shown to increase immediately after weight loss, remaining at almost identical levels 1 year later in the weight-maintenance phase, despite steady and sustained partial weight regain^(^^65^^)^. Our results, however, showed no difference in subjective appetite ratings, in the fasted state and postprandially, between RED and LSW, showing that RED are not at increased risk for weight regain as a result of greater appetite sensations. REL, however, reported greater appetite drive in the fasted state compared with controls, potentially increasing their risk for greater subsequent food intake. However, postprandially they reported greater reductions in appetite drive, while this rises in controls to levels at or above those reported in the fasted state, which may increase the risk for regular snacking in always overweight/obese, despite prior intake of high-fat, energy-dense foods.

Objectively measured physical activity showed that RED engaged in more moderate activity, had a tendency for increased vigorous activity and spent less time in sedentary behaviour. NREE, both planned and spontaneous physical activity, declines during weight loss as well as weight-loss maintenance over the following 6 and 12 months, and is associated with subsequent weight regain^(^^66^^)^. However, other studies have demonstrated that while NREE is reduced during weight loss, it recovers during weight maintenance, and high levels of physical activity are characteristic of successful weight-loss maintainers, which is in line with these results^(^^24^^,^^67^^)^. Consistently maintaining higher levels of moderate and vigorous activity and reducing sedentary behaviour may be key strategies that have assisted the RED group to successfully maintain previous weight loss.

There was no evidence of metabolic inflexibility in RED or REL compared with respective controls, both fasted and in response to the high-fat test meal. RED were significantly more insulin sensitive than LSW, characterised by considerably lower fasting insulin levels (details published previously^(^^68^^)^). Previous studies show that insulin resistance reduces fasting fat oxidation capacity and increases postprandial fat oxidation under insulin-stimulated conditions^(^^69^^)^. With weight loss, improvements in insulin sensitivity result in lower 24 h and postprandial fat oxidation, despite enhanced clearance of dietary fat^(^^31^^,^^70^^–^^72^^)^, suggesting a preference for fat storage over oxidation in the fasted state. Despite being more insulin sensitive, these results show that long-term weight-reduced individuals display similar levels of fasting and postprandial fat oxidative capacity compared with controls with no weight-loss history. Enhanced insulin sensitivity may also promote weight regain through more efficient uptake and utilisation of carbohydrates and storage of dietary fat, with rapid nutrient clearance potentially driving appetite^(^^34^^,^^73^^)^. Protein in particular has long-lasting suppressive effects on appetite hormones, while carbohydrates tend to act more acutely with subsequent rebounds in appetite^(^^74^^,^^75^^)^. Compared with LSW, RED reported greater protein and fat intake and reduced carbohydrate intake, suggesting behavioural adaption that may support appetite control and weight-loss maintenance.

The present study was able to identify individuals who had lost a significant amount of weight and either successfully maintained this weight loss for a median of 30 months or regained all of the weight previously lost. This enabled investigation into the longer-term effects of weight loss and regain compared with individuals without a history of weight loss and regain. Objective measurements of physical activity also avoided issues around over- or under-reporting. However, there were limitations to the study. First, the study was cross-sectional and therefore, in line with aims, can only point to associations and make metabolic and behavioural comparisons against matched controls. Second, it may be argued that the choice of a liquid test meal may have led to lesser effects on both appetite and thermogenesis. However, our study sought to identify evidence of appetite dysfunction in obesity that might remain present in post-obese individuals. This has been identified in other studies where a high-fat pre-load led to subsequent passive overconsumption relative to always-lean individuals, and could potentially contribute to weight-loss relapse^(^^45^^)^. This study relied on self-reported weight history rather than documented weight changes from formal interventions. It also made use of bioelectrical impedance analysis for measurement of body composition, which has limitations. Lastly, we had relatively fewer participants in the REL and OSW groups which may have underpowered the effects shown for these comparisons.

In conclusion, the present study found no differences between reduced-weight individuals and always-lean controls in terms of RMR and substrate oxidation, implying that the reduced-weight individuals are not in a metabolic state that will promote weight regain. This finding contributes to the small body of evidence that not all reduced obese individuals are at a metabolic disadvantage that will promote weight regain^(^^28^^,^^61^^,^^71^^)^. In weight-loss relapsed individuals metabolic parameters and behavioural strategies were indistinguishable from matched controls, showing that weight loss-associated metabolic disadvantage is probably reversed with weight regain. However, weight-reduced individuals were more insulin sensitive, potentially increasing the risk for weight regain with dietary relapse and physical inactivity. They exerted significant conscious behavioural efforts to maintain previous weight loss, reporting greater dietary restraint, manipulating macronutrient components of the diet that supported maintenance of FFM as well as improving satiety and were more physically active compared with controls. Adaptive thermogenesis is shown to accompany weight loss and can be sustained in weight-loss maintenance, reducing all components of TDEE. However, similar metabolic rates shown for components of TDEE in successful weight-loss maintainers compared with controls is probably the result of conscious behavioural strategies that act together to counteract weight loss-associated declines in energy expenditure. This highlights the importance of these strategies as key components in weight-maintenance programmes following substantial weight loss.
